# The Unprecedented Influenza Epidemic of 2024/2025: Overshadowing the COVID‐19 Winter Wave and Exploring Viral Interference

**DOI:** 10.1002/iid3.70300

**Published:** 2025-11-20

**Authors:** Benjamin Davido

**Affiliations:** ^1^ Maladies Infectieuses, Hôpital Raymond‐Poincaré Université Paris Saclay, AP‐HP Garches Ile‐de‐France France; ^2^ UMR1173 Université Versailles Saint‐Quentin Montigny‐Le‐Bretonneux Ile‐de‐France France; ^3^ Mission Ministérielle de la Prévention des infections et de l'antibiorésistance, Direction Générale de la Santé Paris Ile‐de‐France France

**Keywords:** 2024/2025, Covid‐19, Influenza, SARS‐CoV‐2, viral interference

## Abstract

**Background:**

In winter 2024/2025, one of the most severe U.S. influenza epidemics since 2017, with 82 million illnesses and ~ 130,000 deaths, eclipsed a comparatively subdued SARS‐CoV‐2 wave (~ 20 million infections, ~540,000 hospitalizations, ~63,000 COVID‐19 deaths), a hypothesis supported by existing CDC data and virological models.

**Hypothesis:**

Influenza's robust interferon response might have contributed to suppression of SARS‐CoV‐2 replication, creating an antiviral environment that delayed COVID‐19's winter surge.

**Evidence:**

In vitro studies demonstrate influenza interference with SARS‐CoV‐2, reversible by oseltamivir, supporting this hypothesis. Thus, the decline in influenza activity in spring 2025 may have contributed to the observed increase in SARS‐CoV‐2 hospitalizations, driven by the NB.1.8.1 variant, a JN.1 derivative with enhanced transmissibility and reduced neutralization. Although this did not evolve into a pandemic‐scale wave, the resultant burden on healthcare services was clinically meaningful. Alternative explanations, including behavioral changes and Omicron‐induced herd immunity, are considered but less compelling.

**Implication:**

The global health implications are critical: lapsed COVID‐19 preparedness, limited surveillance, and low vaccine uptake threaten effective responses.

**Recommandations:**

We advocate enhanced surveillance, adopting wastewater monitoring as demonstrated in the U.S., and integrated vaccination campaigns with combined influenza‐COVID‐19 vaccines, currently in phase III trials, to boost immunity. Resource allocation, informed by the 2024/2025 hospital overload, is essential to manage surges. This article underscores the need for global coordination to understand viral interference and prepare for shifting respiratory virus dynamics, offering actionable strategies to mitigate future epidemics, including those caused by other viruses.

## Summary

- Severe flu surge hit U.S. in 2024/2025, COVID‐19 cases dropped.
- Viral interference may explain flu dominance over SARS‐CoV‐2.
- Influenza A/H3N2 triggered interferon, hindering COVID‐19 spread.
- CDC: 45 M flu cases, 580K hospitalizations by March 29, 2025.
- More research needed to confirm viral interference mechanism.

## Graphical abstract

In winter 2024/2025, a severe flu surge in the United States, particularly in California and New York, outpaced declining COVID‐19 cases, with viral interference explored as a potential cause. The combined burden of 26,000 flu and 28,000 COVID‐19 deaths underscores the need for a holistic approach to promote vaccination.

## Introduction

Respiratory viruses, including influenza and SARS‐CoV‐2, have historically exhibited seasonal patterns, often leading to increased morbidity and mortality during the winter months. The winter of 2024/2025, however, presented an anomaly: a significant and unpredicted influenza epidemic coupled with an unanticipated decline in COVID‐19 cases. Of note, in contrast to influenza, COVID‐19 case notifications are no longer systematically tracked, but the CDC continues to provide national indicators through hospitalizations, mortality, and wastewater surveillance, complemented by modeling estimates. Indeed, interpreting multi‐seasonal trends must consider that from 2020 to 2022, widespread non‐pharmaceutical interventions (NPIs) dramatically reduced the circulation of seasonal respiratory viruses. Therefore, the most relevant seasons for comparison are 2023/2024 and 2024/2025, as they occurred without mitigation measures. However, this must be interpreted in the broader context of the 2020/2021 and 2021/2022 pandemic seasons, during which population immunity to SARS‐CoV‐2 was still building. By 2024/2025, SARS‐CoV‐2 had become endemic, with population‐level immunity playing a dominant role in shaping viral dynamics. This focus aligns with the World Health Organization's declaration ending the COVID‐19 global public health emergency on 5 May 2023, which formally marked the transition out of the pandemic phase [1].

Understanding the dynamics between co‐circulating respiratory viruses is crucial for public health planning and response strategies, as these cannot rely solely on mathematical modeling. These interactions may involve mechanisms such as viral interference, herd immunity shifts, or behavioral changes, which this article discusses as possible contributing factors rather than as proven mechanisms. Although no pandemic‐like SARS‐CoV‐2 epidemic wave occurred after spring 2025, the cumulative effect of SARS‐CoV‐2, influenza, RSV and other respiratory viruses remains a major concern for vulnerable populations, reinforcing the need for preparedness.

### Epidemiological Overview of the 2024/2025 Winter Season

This analysis focuses on the two most recent influenza seasons (2023/2024 and 2024/2025), as they represent the first truly “post‐pandemic” respiratory seasons. Notably, a SARS‐CoV‐2 wave was already observed in autumn 2024, yet the absence of a typical winter 2024/2025 surge remains unusual occurring in the context of SARS‐CoV‐2's transition from pandemic to endemic circulation. Importantly, an autumn 2024 wave was already observed in the United States and Europe, particularly among individuals with waning neutralizing antibodies or immunity against more distant variants. This two‐peak dynamic in the Northern hemisphere suggests that the apparent anomaly may partly reflect timing and coincidence, rather than viral interference alone. In contrast, the seasons from 2019 to 2022 were deeply confounded by public health interventions and behavioral disruptions.

As May 17th 2025, the 2024/2025 influenza season was marked by one of the most severe influenza activities since 2017 in the United States [2, 3], with the Centers for Disease Control and Prevention (CDC) estimating up to 82 million illnesses, 1.3 million hospitalizations, and 130,000 deaths in contrast with 40 million of cases and 470,000 admissions for 2023/2024 [2]. In comparison, as September 20th 2025 SARS‐CoV‐2 activity remained relatively subdued, with an estimated 20.3 million cases, 540,000 hospitalizations and 63,000 COVID‐19 deaths, (see Table 1) [3]. This divergence from expected seasonal co‐circulation patterns has prompted renewed attention to possible mechanisms of viral interference, where the predominance of one virus may suppress the activity of another [4].

**Table 1:** Respiratory virus burden in the US and EU/EEA, 2023–2025.

Season	US – Influenza	EU/EEA – Influenza	US – COVID‐19	EU/EEA – COVID‐19
2023–24	40 M/470,000 hospitalizations and 28,000 deaths	26,118 cases/8,659 hospitalizations/665 deaths	~5.3 M/ ~ 362,000 hospitalizations and 41,350 deaths	No major winter surge; no excess mortality (EuroMOMO*); monitoring ceased Nov 2023
2024–25	47‐82 M cases/610,000–1.3 M hospitalizations and 27,000–130,000 deaths	354,455 confirmed cases; positivity up to 48.4% (ECDC/ERVISS**)	13.8 ‐20.3 M ilnesses/380,000–540,000 hospitalizations and 44,000–63,000 deaths	Positivity declined to 1.6% during winter; modeled burden ~12,000–24,000 deaths

*Note:* EuroMOMO* European mortality monitoring, EUROMOMO. ERVISS** The European Respiratory Virus Surveillance Summary The European Respiratory Virus Surveillance Summary (ERVISS).

In Europe, data from the European Center for Disease Prevention and Control (ECDC) confirmed a lower burden of disease for influenza. According to the ECDC's Annual Epidemiological Report, 26,118 laboratory‐confirmed influenza cases were reported, with 8,659 hospitalizations and 665 deaths across 26 EU/EEA countries during the 2023/2024 season [5].

**Figure 1:**
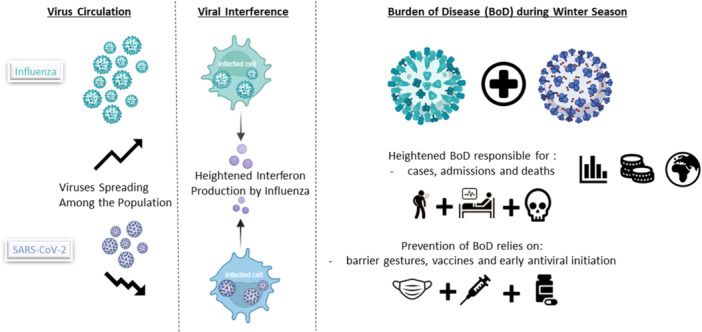
Summarize of the seasonal respiratory virus burden of disease, depicting unprecedented influenza activity in comparison to COVID‐19 trends and illustrating the proposed viral interference between influenza and SARS‐CoV‐2.

Conversely, following the end of the pandemic, SARS‐CoV‐2 surveillance was gradually phased out, with the last national‐level 14‐day notification rate published in November 2023. However, EU‐wide surveillance indicated no major winter surge in 2023/2024, ongoing monitoring through platforms such as ERVISS (European Respiratory Virus Surveillance System) and positivity trends still allow for assessing respiratory virus dynamics [6]. As of early October 2024, pooled EU/EEA positivity rates were 15.8% for SARS‐CoV‐2 and only 1.2% for influenza. By early April 2025, these trends had reversed dramatically with SARS‐CoV‐2 positivity dropping to 2.6% and influenza climbing to 15.6% (Table 1) supporting the hypothesis of divergent dynamics and reinforce the plausibility of a competitive interaction between influenza and SARS‐CoV‐2. This overall pattern is summarized visually in Figure 1.

### Viral Interference: A Plausible Hypothesis

Viral interference refers to the phenomenon where infection with one virus can inhibit the replication or spread of a second virus. This can occur through various mechanisms, including the host's immune response and competition for cellular resources. Historically, instances of viral interference have been observed between influenza and rhinoviruses, suggesting that the presence of one respiratory virus can modulate the activity of another through interferon‐mediated mechanisms [7].

Moreover, mathematical simulations modeling pathogen dynamics have already demonstrated that transient immune‐mediated interference can play a role during seasons when both influenza A virus (IAV) and rhinovirus circulate, supporting the potential influence of innate immunity in driving their asynchronous circulation [8].

These data, generated by other groups, provide context for our discussion rather than constituting direct investigation.

### Influenza Epidemic Limiting COVID‐19 through Viral Interference?

A study by Gilbert‐Girard et al. [9] investigated interactions between SARS‐CoV‐2 and IAV using human airway epithelial models. The findings indicated that influenza A, particularly the A/H3N2 strain but also H1N1, interfered with SARS‐CoV‐2 replication. This interference was attributed to a robust interferon response elicited by the IAV, which SARS‐CoV‐2 alone did not significantly induce. Likewise, Cheemarla et al. [10] demonstrated the same pathway of interference between IAV and SARS‐CoV‐2, while also showing that oseltamivir, an antiviral agent targeting influenza, can restore SARS‐CoV‐2 replication by suppressing influenza replication.

It is noteworthy that bidirectional interference remains possible. For instance, during the 2021/2022 season, widespread Omicron circulation appeared to suppress influenza activity, as SARS‐CoV‐2 outcompeted other respiratory viruses [11]. However, was strongly influenced by NPIs and higher transmissibility of SARS‐CoV‐2 variants, particularly under early pandemic containment conditions. Comparisons across seasons must account for behavioral immunological, and policy‐related confounders.

Moreover, defective interfering particles (DIPs) represent another layer of complexity [12]. These naturally occurring viral genome variants can outcompete standard virus strains within coinfected cells, potentially amplifying interferon production and suppressing secondary infections. Though DIPs are under‐studied in the context of SARS‐CoV‐2 and influenza co‐circulation, future investigations may clarify their contribution to interference phenomena. In addition, recent work has also documented interference phenomena involving SARS‐CoV‐2 and rhinovirus [13], further supporting the plausibility of such interactions, though causality remains difficult to establish.

Taken together, both experimental and population‐level evidence have suggested in prior studies a plausible mechanistic basis for the reduced SARS‐CoV‐2 burden during the 2024/2025 influenza‐dominated season. Here, we summarize and contextualize these observations, rather than presenting new experimental data.

### Alternative Explanations

While viral interference offers a compelling explanation, other plausible mechanisms may have contributed to the observed divergence:

1. Public health measures: Even in the absence of strict NPIs, increased awareness and flu vaccination uptake may have incidentally affected viral transmission dynamics. However, there is no real‐world data to support an unprecedented vaccination campaign this winter, nor the use of face masks as seen during the pandemic.
2. Behavioral changes: The severity of the influenza epidemic may have led to behavioral modifications, such as reduced social interactions, indirectly reducing COVID‐19 spread. A similar phenomenon was observed during 2020, when COVID‐19 mitigation coincided with vanishing influenza incidence [14].
3. Herd immunity: Following repeated Omicron waves, particularly driven by JN.1 and related subvariants, the population acquired a higher degree of hybrid immunity against KP.3 and XEC [15]. This immunity was not limited to neutralizing antibodies, but also involved broader protective mechanisms (opsonisation, complement activation, ADCC, T cell responses), which likely contributed to the absence of a major spring 2025 SARS‐CoV‐2 wave. Nevertheless, waning immunity may permit local resurgences, particularly when driven by variants with reduced neutralization.
4. Complex interactions between other underestimated viruses in circulation: Although influenza and SARS‐CoV‐2 typically peak in winter, they are not strictly seasonal viruses. Other paramyxoviruses, such as respiratory syncytial virus, parainfluenza virus and metapneumovirus [16], may have played a background role in modifying the interplay between influenza and SARS‐CoV‐2.

### Resurgence of COVID‐19 in Spring 2025 With Variant NB.1.8.1

The unusual summer surge of SARS‐CoV‐2 infections in 2024, driven by Omicron sublineages such as KP.3 and XEC, persisted into late fall, likely delaying the anticipated winter surge. Recent evidence indicates that the NB.1.8.1 variant, a derivative of JN.1, has driven a resurgence of COVID‐19 cases and admissions in spring 2025, characterized by enhanced transmissibility and potential immune evasion [17]. NB.1.8.1's higher ACE2 binding affinity, as observed in JN.1, contributes to its dominance and persistence in human populations. The decline in influenza activity by spring 2025 may have created a window for SARS‐CoV‐2 to rebound, as the interferon‐mediated antiviral environment wanes.

### Implications for Public Health

Understanding respiratory virus interference is essential for pandemic preparedness and seasonal vaccination strategies:

- Surveillance: Continuous monitoring through platforms like ERVISS and wastewater sequencing [18, 19] is essential to anticipate and manage potential interference effects.
- Vaccination Strategies: The integration of influenza and COVID‐19 vaccine platforms, especially through dual‐component candidates currently in phase III trials, may increase uptake and confer broader community immunity [20].
- Hospital Readiness: Recognizing patterns of viral dominance can inform healthcare resource distribution, ensuring preparedness for surges in specific infections [21].

### Current Evidence and Research Gaps

While the concept of viral interference is biologically plausible, establishing causation for viral interference remains challenging. Most supporting data are in vitro or ecological. Expanded immunovirological studies during real‐world co‐circulation periods are needed, along with modeling of competing seasonal viruses.

We do not claim viral interference is the sole driver of seasonal patterns; instead, we propose it as a plausible, biologically supported contributor that, together with immunity, behavior and surveillance dynamics, informs preparedness strategies such as integrated vaccination and enhanced pathogen monitoring.

Indeed, the emergence of NB.1.8.1, a JN.1 derivative, underscores the need for enhanced genomic surveillance to track variant evolution and its impact on viral interference dynamics, particularly as shifting respiratory virus patterns may influence the 2025/2026 season.

## Conclusion

The severe influenza activity of 2024/2025 and the relative quiescence of SARS‐CoV‐2 support the possibility of viral interference, further strengthened by supportive epidemiological and experimental evidence. However, a multifactorial explanation encompassing immunity, behavioral adaptation, and surveillance gaps must be acknowledged. To anticipate future waves, it is critical to invest in integrated surveillance, next‐generation vaccines, and adaptive public health strategies.

## Author Contributions

**Benjamin Davido:** conceptualization, validation, writing – review and editing, visualization.

## Conflicts of Interest

The author declares no conflicts of interest.

## Data Availability

The author has nothing to report.
